# Epigenetic profiling of preterm birth: a dual-tissue methylation patterns using long-read sequencing

**DOI:** 10.3389/freae.2025.1644521

**Published:** 2025-12-05

**Authors:** BalaSubramani Gattu Linga, Faisal E. Ibrahim, Aleem Razzaq, Muthanna Samara, Jameela Roshanuddin, Hind H. Adi, Aseel Al-Dewik, Ayla J. Ahmedoglu, Rand Hamdan, Amal E. I. Ahmed, M. Walid Qoronfleh, Hatem Zayed, Duaa Elshiekh, Mona Ellaithi, Mohamed Alsharshani, Palli Valapila Abdulrouf, Thomas Farrell, Bader Kurdi, Ghassan Abdoh, Hilal Al Rifai, Nader Al-Dewik

**Affiliations:** 1 Department of Research, Women’s Wellness and Research Center, Hamad Medical Corporation, Doha, Qatar; 2 Translational and Precision Medicine Research Facility,Women’s Wellness and Research Center (WWRC), Hamad Medical Corporation (HMC), Doha, Qatar; 3 Translational Research Institute (TRI), Hamad Medical Corporation (HMC), Doha, Qatar; 4 Department of Psychology, Kingston University London, London, United Kingdom; 5 Qatar Preterm and Precision Medicine Research Clinic, Women’s Wellness and Research Center, Hamad Medical Corporation (HMC), Doha, Qatar; 6 Health Profession Awareness Program, Health Facilities Development, Hamad Medical Corporation (HMC), Doha, Qatar; 7 School of Medicine, Bahcesehir University, Istanbul, Türkiye; 8 Department of Biological and Environmental Sciences, College of Arts and Sciences, Qatar University, Doha, Qatar; 9 Health Research & Policy Division, Q3 Research Institute (QRI), Ann Arbor, MI, United States; 10 Biomedical Research Center, Qatar University, Doha, Qatar; 11 Faculty of Medical Laboratory Sciences, Al-Neelain University, Khartoum, Sudan; 12 Diagnostic Genetics Division (DGD), Department of Laboratory Medicine and Pathology (DLMP), Hamad Medical Corporation (HMC), Doha, Qatar; 13 Clinical Trial Unit, Academic Health System - Hamad Medical Corporation, Doha, Qatar; 14 Department of Obstetrics and Gynecology, Women’s Wellness and Research Centre, Hamad Medical Corporation, Doha, Qatar; 15 Faculty of Medicine, Qatar University, Doha, Qatar; 16 Neonatal Intensive Care Unit (NICU), Newborn Screening Unit, Department of Pediatrics and Neonatology, Women’s Wellness and Research Center (WWRC), Hamad Medical Corporation (HMC), Doha, Qatar; 17 Genomics and Precision Medicine (GPM), College of Health & Life Science (CHLS), Hamad Bin Khalifa University (HBKU), Doha, Qatar; 18 Faculty of Health and Social Care Sciences, Kingston University, St. George’s University of London, London, United Kingdom

**Keywords:** preterm birth (PTB), DNA methylation, long-read sequencing, epigenetic biomarkers, maternal-fetal interface, methylome, third-generation sequencing

## Abstract

**Introduction:**

Preterm birth (PTB), a leading cause of neonatal morbidity and mortality, arises from complex maternal-fetal interactions with multifactorial origins. Emerging evidence suggests that epigenetic dysregulation may mediate these interactions. This study aimed to identify DNA methylation changes associated with PTB to uncover potential biomarkers and underlying mechanisms.

**Methods:**

We employed long-read sequencing to profile genome-wide DNA methylation followed by gene ontology and pathway enrichment analysis in matched maternal peripheral blood and neonatal cord blood from 15 preterm and 7 full-term deliveries (mother–infant pairs).

**Results:**

A total of 1,151 significantly differentially methylated regions (DMRs) and 25,336 differentially methylated loci (DMLs) were identified across maternal and neonatal blood samples. In maternal blood from PTB cases, the most significantly hypermethylated genes were *MED38*, *PSMB11*, and *WNT7B*, whereas *EXTL3* and *MMP9* were among the most hypomethylated. Additionally, the promoters of *VWA5A*, *EIF4E3*, *ZNF571*, and *COPB2* exhibited significant hypermethylation, while those of *SIRPB1* and *TNFRSF19* showed hypomethylation. In neonatal cord blood from PTB cases, the most significantly hypermethylated genes were *LOC401478*, *ISG20*, *LMTK3*, *TCAF2*, and *COL4A2*, whereas *EXTL3* and *MMP9* were among the most hypomethylated. Promoters of *DKK3*, *CELF2*, and *IFI35* were notably hypermethylated, whereas *ALOX12* and *CLBA1* were among the most hypomethylated. Enrichment analysis revealed that these epigenetic alterations impact critical developmental, immune, and neuroendocrine pathways, including Wnt signaling, calcium signaling, MAPK, oxytocin signaling, and neuroactive ligand-receptor interaction. Comparative analysis identified 120 overlapping DMLs, with 91 hypermethylated and 28 hypomethylated consistently across maternal and neonatal samples, including *DPPA3*, *ABCA1*, and *GKN1*. In contrast, 20,240 and 4,770 DMLs were unique to cord and peripheral blood, respectively. Additionally, 14 overlapping DMRs were mapped to genes such as *PLD5*, *FBXO40*, *GMNC*, *HHIP*, *CLEC18B*, and *LHX1*, exhibiting non-random chromosomal clustering. Enrichment analysis of these shared DMRs revealed significant involvement in developmental processes, including skeletal morphogenesis, axis patterning, and fibroblast growth factor signaling, indicating convergence on core regulatory pathways in PTB.

**Conclusion:**

This is the first dual-tissue PTB study using long-read methylation profiling. Our results reveal distinct and shared epigenetic signatures in maternal and neonatal compartments, offering insights into the molecular etiology of PTB and potential biomarkers for early detection and therapeutic intervention.

## Graphical Abstract

**Figure:**
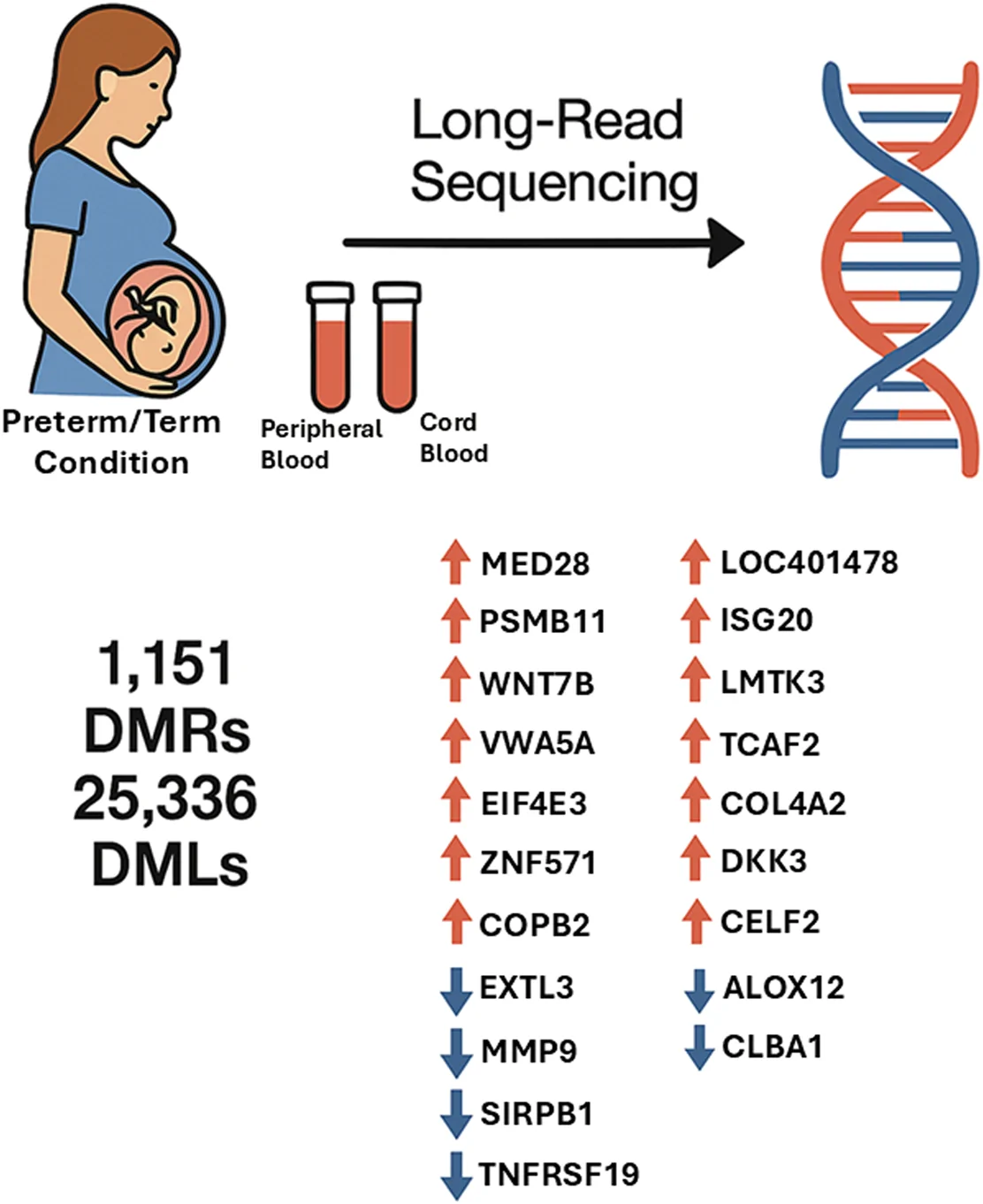

## Introduction

Preterm birth (PTB) – delivery before 37 weeks’ gestation–remains a pressing global health issue. Approximately 15 million infants (about 10% of all births) are born preterm each year, with PTB complications responsible for roughly one million deaths annually (Walani, 2020; World Health Organization, 2023). PTB is the leading cause of neonatal and under-five child mortality worldwide, accounting for ∼35% of newborn deaths (Walani, 2020). PTB rates vary globally: 10.41% in the United States in 2023; across Europe, country-specific rates in 2019 ranged from 5.3% to 11.3% (median approximately 6.9%); and in low- and middle-income countries (LMICs), particularly in low-income settings, survival among infants born before 28 weeks remains less than 10% (Osterman et al., 2025; Euro-Peristat Research Network, 2022; World Health Organization, 2023). In comparison, the incidence of preterm birth (PTB) and early term birth (ETB) in Qatar is 8.8% and 33.7%, respectively. Early PTB, defined as delivery before 32 weeks of gestation, comprises 33.7% of all preterm cases, while ETB—defined as delivery between 37 and 38 weeks - represents a substantial proportion of term births (Younes et al., 2021). Younes et al. (2021) PTB affects 5%–10% of pregnancies and contributes substantially to perinatal morbidity and mortality in both developed and developing regions. Notably, 85% of neonatal deaths in non-anomalous infants are due to PTB (Wang et al., 2022). Male infants represent over half of preterm births and have higher neonatal mortality rates than females. Long-term complications of PTB include neurodevelopmental impairments, cerebral palsy, and chronic respiratory illness (Pidsley et al., 2013; Razzaq et al., 2025). The rising incidence of PTB, especially in industrialized nations, persists despite advances in understanding risk factors and public health interventions aimed at its prevention (Goldenberg et al., 2008).

The etiology of PTB is multifactorial, involving a complex interplay of maternal, fetal, genetic, environmental, and epigenetic factors that can trigger preterm labor. Risk factors span a broad spectrum: from non-modifiable contributors like a prior history of PTB, multifetal pregnancy, uterine or cervical anomalies, and extreme maternal age, to modifiable exposures such as infection, inflammation, poor nutrition, obesity, smoking, socioeconomic deprivation, and chronic stress (Samuel et al., 2019). Despite this, up to 50% of PTB cases occur without a clearly identifiable cause, underscoring the need to investigate additional biological mechanisms (Muglia and Katz, 2010; Samuel et al., 2019). To bridge the gap between diverse risk factors and biological outcomes, epigenetic mechanisms, particularly DNA methylation, have emerged as promising mediators in PTB (Camerota et al., 2024). DNA methylation, the addition of methyl groups to CpG sites in DNA, plays a pivotal role in gene regulation and fetal development and is highly responsive to environmental exposures (Oberlander et al., 2008; Conradt et al., 2016; Lester et al., 2018). These epigenetic modifications are stable yet reversible, making them attractive targets for mechanistic and therapeutic investigation. Methylation profiles may reflect cumulative influences such as maternal infection, stress, or poor nutrition during pregnancy. Recent studies have associated aberrant DNA methylation patterns with PTB (Zhang et al., 2017; Hong et al., 2018; Wang et al., 2019). For instance, epigenome-wide association studies (EWAS) have revealed PTB-associated differentially methylated loci (DMLs) in maternal blood, especially in genes related to inflammation and hormone signaling, which were not mirrored in the corresponding fetal cord blood (Hong et al., 2018). Similarly, methylation profiling of placental tissue and neonatal blood revealed PTB-associated changes in both compartments but with minimal overlap, suggesting tissue-specific epigenetic signatures (Wang et al., 2019). Notably, the genes showing differential methylation in placenta were enriched for immune and developmental pathways, whereas those in cord blood were involved in neurodevelopment, highlighting how PTB’s epigenetic imprint can vary by tissue (Wang et al., 2019). Placental DMRs were enriched in immune and developmental pathways, while neonatal changes implicated neurodevelopmental genes.

However, most previous PTB methylation studies have been limited by small sample sizes and the use of a single tissue–maternal blood, placenta, or cord blood–analyzed in isolation. This narrow focus may contribute to inconsistent findings across studies (Hong et al., 2018; Wang et al., 2019). Technically, many prior studies relied on array-based platforms or short-read bisulfite sequencing (e.g., Illumina 450K/850K arrays), which profile only a small fraction of the genome. These methods lack the resolution to detect methylation in repetitive or intergenic regions and cannot resolve haplotype-specific methylation or long-range epigenetic interactions (Fan et al., 2016; Pidsley et al., 2016; Wang et al., 2018). These limitations restrict the ability to comprehensively assess the methylome and to detect subtle, biologically significant changes associated with PTB.

Recent advances in long-read DNA sequencing offer a transformative solution to these challenges. Platforms like Oxford Nanopore can generate kilobase-length reads from native (non-bisulfite-converted) DNA, allowing direct detection of cytosine methylation at base-pair resolution. Compared to array-based approaches, long-read technologies interrogate orders of magnitude more CpG sites and enable near-complete methylome coverage (Logsdon et al., 2020). Crucially, long reads permit phasing of methylation patterns across individual DNA molecules, revealing allele-specific and co-methylation features that short-read technologies cannot resolve (Logsdon et al., 2020). These platforms also excel in accessing GC-rich and repetitive regions, enhancing detection of epigenetic variation in previously unresolvable genomic contexts. Together, these capabilities position long-read methylation sequencing as a powerful tool for elucidating the epigenetic underpinnings of complex disorders like PTB.

In this study, we leverage long-read sequencing to perform the first dual-tissue methylation analysis (maternal peripheral blood and neonatal cord blood) in the context of preterm birth. Using Oxford Nanopore sequencing, we profile the DNA methylation landscapes in matched maternal and fetal samples from preterm and term deliveries at single-base resolution. This approach allows for unbiased, high-coverage methylation detection without bisulfite conversion and enables integration of genetic and epigenetic variation. We aim to uncover robust, tissue-specific epigenetic alterations associated with spontaneous PTB by analyzing methylation patterns across both compartments. This comparative dual-tissue methylation analysis using long-read technology between preterm birth (PTB) cases and full-term birth (FTB) controls aims to enhance our understanding of the maternal-fetal epigenetic interface in PTB and to identify potential biomarkers for prediction and intervention.

## Materials and methodology

### Study design and participants

This hospital-based case–control study was conducted at the Women’s Wellness and Research Center (WWRC), Qatar, from September 2022 to December 2024. The inclusion criteria comprised pregnant women who delivered either preterm (<37 weeks of gestation) or full-term (≥37 weeks of gestation). Only singleton pregnancies were considered in both groups, and neonates with congenital anomalies were excluded. The study included 22 singleton mother-infant pairs who had natural labor, comprising 15 preterm births (gestational age of less than 32 weeks) and seven full-term births (gestational age of 37-42 weeks) with normal birth weight.

### Biological sample collection and DNA extraction

A volume of 5 mL of of maternal peripheral blood samples using EDTA tubes was collected from mothers prior to or at the same time as delivery, and 5 mL of neonatal cord blood using EDTA tubes was collected immediately after the newborn was delivered. Peripheral and cord blood DNA were extracted using the Maxwell DNA Blood Kit (Promega, United States) following the manufacturer’s instructions. The DNA was eluted in a 30 μL elution volume. Extracted DNA samples were quantified by fluorometric analysis (Qubit™ dsDNA HS Assay Kit, Invitrogen, United States) and visualized on 1% agarose gels. The A260/A280 ratio was measured with a NanoDrop to confirm DNA purity. Results were in the range of 1.8–2.1.

### Library preparation and nanopore sequencing

Sequencing libraries were generated using the SQK-LSK109 ligation kit from ONT using 1 μg DNA. Samples were loaded onto R10.4.1 flowcells following ONT standard operating procedures. The sequencing was performed on PromethION devices**.**

### Basecalling and mapping

Basecalling was performed using Dorado v6.2.7 on sequencing data generated with R10.4 flowcells (Dorado, 2023). This tool converts raw nanopore signal data into nucleotide sequences while concurrently detecting CpG methylation. Dorado leverages neural network-based model to improve accuracy, particularly in identifying base modifications. CpG methylation tags were extracted from the unaligned BAM files produced during basecalling and subsequently integrated into the aligned BAM files for downstream methylation analysis. A total of 44 samples were sequenced, each on an individual R10.4 flowcell. Basecalling, alignment, and CpG methylation detection were all executed locally using Dorado v6.2.7 (Dorado, 2023).

### Methylation calling

We employed the EPI2ME Lab MODKIT tool to analyze DNA methylation patterns, a comprehensive pipeline designed to process nanopore sequencing data for methylation analysis. The aligned BAM files were processed using the EPI2ME MODKIT methylation detection pipeline. This step involves detecting base modifications (such as 5mC and 5hmC) directly from the nanopore signal data. MODKIT uses a neural network model to detect methylated cytosines based on deviations in the nanopore signal that are characteristic of base modifications. After methylation calling, the methylation frequency at each CpG site was calculated. The methylation data were then grouped by sample type (e.g., preterm and full-term).

### Methylation data processing and analysis

A structured preprocessing strategy was employed to extract essential genomic features from methylation bed files. Specifically, chromosome identifiers (chr), genomic positions (pos), total read counts (N), and methylated read counts (X) were isolated to enable standardized downstream analyses. Data extraction was performed using automated text-parsing tools to efficiently select and format the required columns into tab-separated values (TSV) files. Following extraction, rigorous quality control checks were conducted to verify column integrity, formatting consistency, and adherence to input requirements for subsequent computational workflows. This preprocessing approach ensured the reproducibility, scalability, and accuracy of methylation data handling, thereby facilitating robust and efficient downstream bioinformatic analyses.

### Differential methylation analysis in cord blood and peripheral blood samples

#### Data acquisition and preprocessing

Methylation data from PTB and FTB samples were extracted and loaded into the R environment (R Core Team, 2024). Data were structured into BSseq objects (Hansen et al., 2012) to serve as input for locus- and region-level methylation analysis.

#### Identification of differentially methylated regions (DMRs) and loci (DMLs)

Differentially methylated regions (DMRs) were identified using the DMRcate package (Peters et al., 2015), which aggregates spatially correlated CpGs into contiguous regions based on methylation differences. Candidate DMRs were filtered using a p-value threshold of <0.01 and a false discovery rate (FDR) cutoff of 0.01. Regions were classified as hypermethylated or hypomethylated based on the mean methylation difference between PTB and FTB samples. DMRs were identified using the DMRcate package, applying a minimum threshold of 3 CpG sites per region (min.cpgs = 3). The average DMR length was 1,191 base pairs in peripheral blood and 1,760 base pairs in cord blood, reflecting the spatial extent of methylation clustering across the genome. DML and DMR identification was based on group-wise comparisons between PTB and FTB samples, performed separately within the peripheral blood (PB) and cord blood (CB) compartments. These analyses were not conducted using pair-wise matching of maternal–fetal sample pairs. Instead, group-level methylation differences were assessed between all PTB and FTB samples within each tissue, adjusting for relevant covariates. No additional filter was applied to require that DMRs or DMLs be shared among a minimum number of individuals. Differentially methylated loci (DMLs) were then identified using the DSS package by fitting a multi-factorial regression model (Wu et al., 2013; Feng et al., 2014; Wu et al., 2015; Park and Wu, 2016). Covariates included sample type (PTB vs. FTB), gestational age, maternal age, and infant sex. The DMLtest.multiFactor function was used to detect significant methylation differences, applying FDR correction for multiple testing. Logistic transformation of methylation levels was performed, and Δ-methylation values were computed. Significant DMLs (FDR <0.01, Δ-methylation >0.1) were retained for further analysis.

### Statistical analysis

Statistical tests were used to assess the significance of differential methylation. A threshold of p < 0.01 was applied for DML detection, and an FDR <0.01 cutoff was used for DMR analysis. Adjustment for covariates such as sample type (PTB vs. FTB). Gestational age, and infant gender was incorporated into all differential analyses. Only loci and regions meeting significance thresholds were included in downstream functional interpretation.

### Annotation of differentially methylated loci and regions using HOMER

Differentially methylated loci (DMLs) and regions (DMRs) from cord blood and peripheral blood were annotated using the HOMER (Hypergeometric Optimization of Motif EnRichment) package. BED-formatted files containing chromosomal coordinates and strand information were input into HOMER to map methylation changes to genomic features, including promoters, exons, introns, intergenic regions, and transcription start sites (TSS). Annotated outputs were analyzed in R to summarize the distribution of methylation across genomic elements.

### Functional enrichment analysis of DMR- and DML-associated genes

Functional enrichment analyses were performed separately for DMR- and DML-associated genes in peripheral and cord blood samples to assess the biological relevance of methylation changes. Gene Ontology (GO) enrichment for Biological Processes and Kyoto Encyclopedia of Genes and Genomes (KEGG) pathway enrichment were conducted using the clusterProfiler package. Enrichment results highlighted the top biological processes and signaling pathways implicated by differential methylation. An adjusted p-value threshold of <0.05 was applied following multiple testing correction. This approach enabled the identification of key developmental, neurological, and signaling pathways potentially affected by epigenetic alterations in preterm birth.

### Overlap and tissue-specific methylation analysis

To investigate shared and tissue-specific methylation alterations associated with PTB, DMLs from peripheral and cord blood samples were compared. Overlapping and tissue-specific DMLs were quantified using Venn diagrams, and delta-beta distributions were assessed. Overlapping DMRs were identified and visualized through hierarchical clustering heatmaps based on chromosomal locations and gene annotations. Gene Ontology (GO) enrichment analysis for Biological Processes was performed using the clusterProfiler R package.

## Results

### Clinical overview of the study cohort

The study included 15 PTB neonates (<32 weeks’ gestation) and 7 FTB neonates, along with their corresponding maternal peripheral blood samples. PTB neonates exhibited a high burden of complications, with neonatal respiratory distress syndrome (NRDS) observed in 13 cases, low birth weight in 6, and extreme prematurity in 8. Additional conditions included neonatal sepsis, hyperbilirubinemia, hypoglycemia, anemia, bronchopulmonary dysplasia, and isolated infectious complications such as Group B *Streptococcus* and *Staphylococcus aureus* colonization. Maternal profiles in the PTB group revealed conventional risk factors for preterm birth. Premature rupture of membranes (PROM/PPROM) was the most frequent complication (n = 8), followed by diabetes mellitus (gestational or type 2) in six mothers, obesity in three, and cervical insufficiency in three cases, with cervical cerclage performed in one. Other maternal conditions included anemia, urinary tract and chorioamnionitis infections. In contrast, FTB neonates and their mothers showed minimal or no clinical abnormalities, with only isolated cases of gestational diabetes or GBS colonization.

### Differentially methylated regions and loci

A total of 1,151 significantly differentially methylated regions (DMRs) and 25,336 differentially methylated loci (DMLs) were identified across maternal and neonatal blood samples. In peripheral blood, 290 DMRs were identified, comprising 124 exonic gene-associated and 166 promoter-associated regions, along with 4,976 DMLs. In cord blood, 861 DMRs were identified, including 409 gene-associated and 452 promoter-associated regions, alongside 20,360 DMLs. Chromosomal mapping of DMRs revealed distinct distribution patterns across autosomes and sex chromosomes (Supplementary Figure S1).

### Differentially methylated regions (DMRs)

#### Peripheral blood

In peripheral blood samples, of the 124 differentially methylated exonic regions, 109 genes were hypermethylated, and 15 were hypomethylated in the PTB cases (Supplementary Table S1). The most significantly hypermethylated genes were *MED28*, *PSMB11*, and WNT7B (−log_10_ p >7.5), while EXTL3 and MMP9 were among the most significantly hypomethylated genes (−log_10_ p >7.5) as illustrated in the Manhattan plot (Figure 1a; Table 1). The genomic distribution of differentially methylated genes (DMGs) in peripheral blood samples is visualized in the circos plot, illustrating the chromosomal localization of both hypermethylated and hypomethylated genes associated with preterm birth (Supplementary Figure S2a). A distinct clustering pattern was observed, with several chromosomal regions showing a higher density of differentially methylated genes in PTB cases compared to term controls. The accompanying heatmap demonstrates clear segregation of preterm and term samples based on methylation profiles of the top-ranked DMGs. These patterns indicate that peripheral blood from PTB cases exhibits coordinated epigenetic alterations affecting multiple genomic regions, supporting a role for widespread methylation reprogramming in preterm birth (Supplementary Figure S2b). Of the 166 differentially methylated promoter regions, 109 were hypermethylated, and 64 were hypomethylated in the PTB cases (Supplementary Table S2). The most significantly hypermethylated genes were *VWA5A*, *EIF4E3*, *ZNF571* and *COPB2* (−log_10_ p >7.5), while *SIRPB1* and *TNFRSF19*, with *SIRPB1* were among the most significantly hypomethylated promoters (−log_10_ p >7.5) (Table 1; Figure 1a).

**FIGURE 1:**
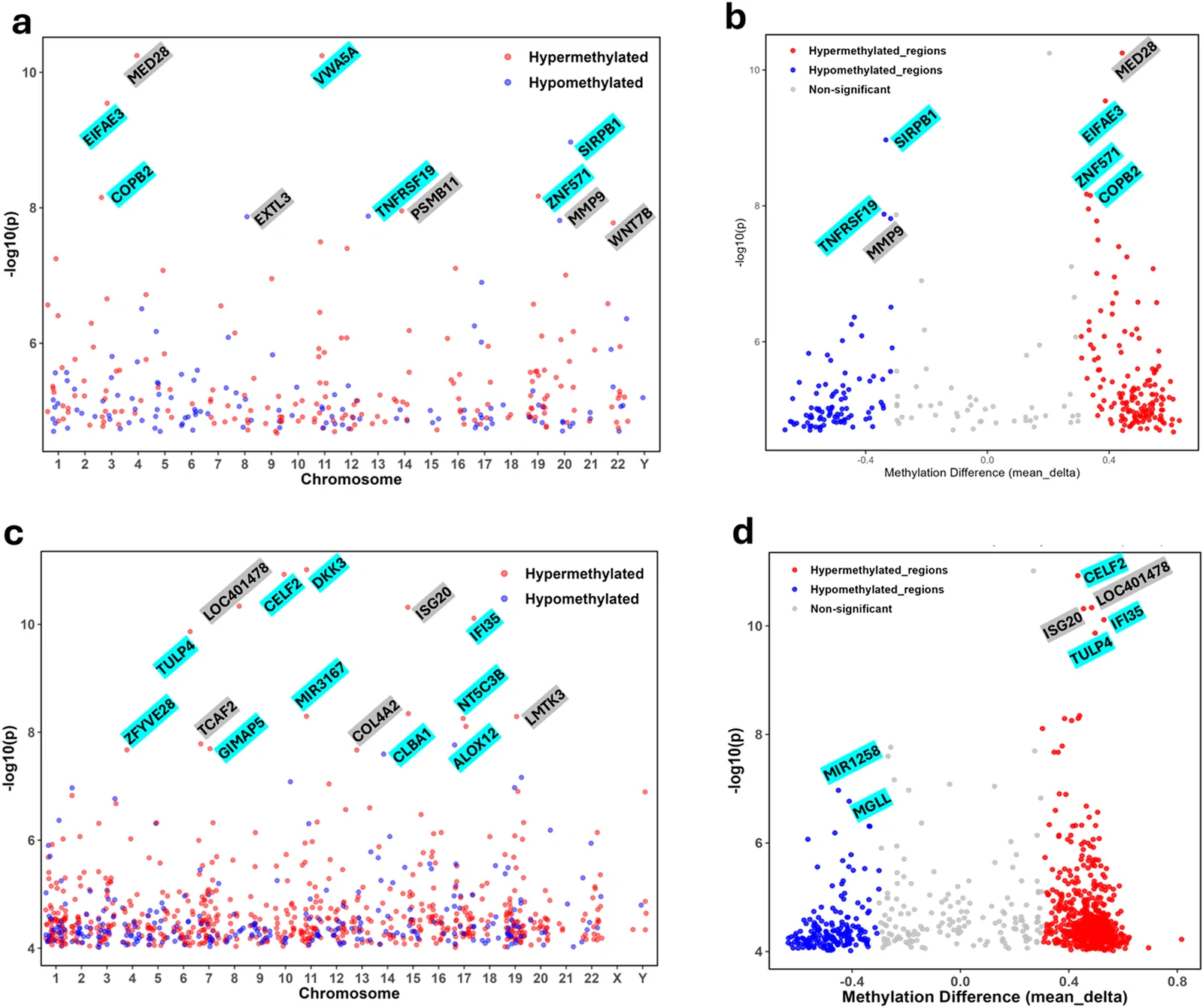
Differentially methylated regions (DMRs) in peripheral and cord blood comparing preterm birth (PTB) and full-term birth (FTB). **(a)** Manhattan plot illustrating differentially methylated regions (DMRs) in peripheral blood across chromosomal locations (x-axis). Statistical significance is represented as −log_10_(p-value) on the y-axis. Red dots indicate hypermethylation in PTB samples, while blue dots indicate hypomethylation. Genes with highly significant methylation changes [−log_10_(p) >7.5] include *EIF4E3*, *MED28*, *VWA5A*, *COPB2*, *EXTL3*, *TNFRSF19*, *PSMB1*, *ZNF571*, *MMP9*, *SIRPB1*, and *WNT7B*. **(b)** Volcano plot depicting DMRs in peripheral blood. The x-axis represents the methylation difference (mean_delta), with hypermethylated loci in red and hypomethylated loci in blue. Statistically significant DMRs [−log_10_(p) >7.5] with strong methylation differences (|mean_delta| >0.3) include *MED28*, *EIF4E3*, and *ZNF571* [−log_10_(p) >8.0, hypermethylated] and *COPB2*, *SIRPB1*, *TNFRSF19*, and *MMP9* [−log_10_(p) >7.5, hypomethylated]. **(c)** Manhattan plot of DMRs in cord blood, with each CpG site positioned by chromosomal location. Red and blue dots indicate hypermethylation and hypomethylation, respectively. Genes with notable methylation differences include TULP4, *LOC401478*, *CELF2*, *DKK3*, *LMTK3*, *ISG20*, *MIR3167*, *IFI35*, *NT5C3B*, *TCAF2*, and *CCDC144CP* [hypermethylated, −log_10_(p) >8] and *MIR1258*, *CLBA1* and *ALOX12* [hypomethylated, −log_10_(p) >7]. **(d)** Volcano plot depicting DMRs in cord blood, with the x-axis representing methylation differences (mean_delta). Red dots indicate hypermethylation, and blue dots indicate hypomethylation. Statistically significant DMRs with large methylation differences (mean_delta >0.3) include *CELF2*, *LOC401478*, *ISG20*, *IFI35*, and *TULP4* [−log_10_(p) >8, hypermethylated] and *MIR1258* and *MGLL* [−log_10_(p) >7.0, hypomethylated]. Genes highlighted in teal represent exonic regions, while those highlighted in turquoise indicate promoter regions.

**TABLE 1:** Top differentially methylated regions (DMGs and DMPs) identified in peripheral blood samples from preterm birth (PTB) cases compared to full-term birth (FTB) controls.

Gene symbol	Methylation status	Annotation	mean_delta	mu_ptb	mu_ftb	log10p_val
*MED28*	Hypermethylated	Exon	0.442	0.581	0.146	10.249
*PSMB11*	Hypermethylated		0.331	0.540	0.306	7.954
*WNT7B*	Hypermethylated		0.359	0.546	0.196	7.777
*EXTL3*	Hypomethylated		−0.299	0.447	0.698	7.868
*MMP9*	Hypomethylated		−0.317	0.561	0.869	7.812
*VWA5A*	Hypermethylated	Promoter	0.203	0.530	0.170	10.247
*EIF4E3*	Hypermethylated		0.387	0.540	0.159	9.544
*ZNF571*	Hypermethylated		0.325	0.494	0.270	8.172
*COPB2*	Hypermethylated		0.338	0.505	0.271	8.152
*SIRPB1*	Hypomethylated		−0.333	0.552	0.226	8.971
*TNFRSF19*	Hypomethylated		−0.339	0.436	0.759	7.877

The table includes both exonic gene-associated regions and promoter regions. Each entry lists the gene symbol, methylation status (Hypermethylated or Hypomethylated in PTB), genomic annotation (exon or promoter), mean methylation difference (mean_delta), average methylation levels in PTB (mu_ptb) and FTB (mu_ftb) groups, and the statistical significance (–log10p value). Positive mean_delta values indicate hypermethylation in PTB, while negative values represent hypomethylation.

To visualize the chromosomal distribution of these promoter-level methylation changes, a Circos plot was generated, illustrating the locations of differentially methylated promoters across all autosomes (Supplementary Figure S2c). This visualization highlights that both hypermethylation and hypomethylation events in PTB are distributed across multiple chromosomes. Promoter methylation profiles in maternal peripheral blood revealed clear distinctions between preterm birth (PTB) and term groups (Supplementary Figure S2d). The heatmap highlighted that *VWA5A*, *PTPRH*, *MIR10522*, and *SLC6A12* exhibited hypermethylated promoter regions in PTB (methylation difference value of >0.55), while *RNF213*, *TNFRSF19*, *SDK1* and *TUBGCP6* (methylation difference value of <0.35) were prominent among the hypomethylated promoter regions. Hierarchical clustering of samples based on promoter methylation patterns revealed two well-separated clusters, corresponding to PTB and term groups, respectively (Supplementary Figure S2d). The clustering pattern underscores consistent epigenetic alterations at the promoter level that distinguish maternal samples associated with PTB. The volcano plot highlighted *MED28*, *EIF4E3*, *ZNF571* and *COPB2* among the most hypermethylated exonic regions with high methylation difference in PTB cord blood, while *SIRPB1*, *TNFRSF19*, and *MMP9* were prominent among the hypomethylated regions (Figure 1b).

#### Cord blood

Of the 409 differentially methylated exonic regions, 365 genes were hypermethylated, and 44 were hypomethylated in the PTB cases (Supplementary Table S3). The most significantly hypermethylated genes were *LOC401478*, *ISG20*, *LMTK3*, *TCAF2* and *COL4A2* (−*log*_*10*_ *p* >7.5) while no hypomethylated exonic regions were identified at the same threshold (−*log*_*10*_ *p >*7.5) (Figure 1c; Table 2). The genomic distribution of differentially methylated genes (DMGs) in cord blood is visualized in a circos plot, illustrating their chromosomal localization and methylation status in PTB versus term cases (Supplementary Figure S3a). The corresponding heatmap highlights the top differentially methylated genes, demonstrating distinct methylation patterns segregating preterm and term samples (Supplementary Figure S3b). Of the 452 differentially methylated promoter regions, 232 were hypermethylated, and 220 were hypomethylated in the PTB cases (Supplementary Table S4). The most significantly hypermethylated genes were *DKK3*, *CELF2*, *IFI35*, *TULP4*, *MIR3167*, *NT5C3B*, *GIMAP5* and *ZFYVE28* (−*log*_*10*_ *p* >7.5), while *ALOX12*, *CLBA1*, *TMEM238*, and *ADO* were among the most significantly hypomethylated promoters (−*log*_*10*_ *p* >7) (Figure 1c) (Table 2).

**TABLE 2:** Top differentially methylated regions (DMGs and DMPs) identified in cord blood samples from preterm birth (PTB) cases compared to full-term birth (FTB) controls.

Gene symbol	Methylation status	Annotation	mean_delta	mu_ptb	mu_ftb	log10p_val
*LOC401478*	Hypermethylated	Exon	0.483	0.579	0.093	10.336
*ISG20*	Hypermethylated		0.453	0.556	0.134	10.317
*LMTK3*	Hypermethylated		0.384	0.630	0.070	8.291
*TCAF2*	Hypermethylated		0.375	0.216	0.666	7.786
*COL4A2*	Hypermethylated		0.360	0.441	0.152	7.671
*DKK3*	Hypermethylated	Promoter	0.269	0.612	0.045	11.013
*CELF2*	Hypermethylated		0.433	0.548	0.076	10.926
*IFI35*	Hypermethylated		0.529	0.633	0.048	10.113
*TULP4*	Hypermethylated		0.496	0.566	0.067	9.866
*MIR3167*	Hypermethylated		0.434	0.591	0.131	8.295
*NT5C3B*	Hypermethylated		0.302	0.511	0.280	8.108
*GIMAP5*	Hypermethylated		0.273	0.601	0.091	7.695
*ZFYVE28*	Hypermethylated		0.345	0.537	0.201	7.673
ALOX12	Hypomethylated		−0.257	0.535	0.213	7.764
CLBA1	Hypomethylated		−0.266	0.539	0.075	7.596

The table includes both exonic gene-associated regions and promoter regions. Each row reports the gene symbol name, methylation status (Hypermethylated or Hypomethylated in PTB), genomic annotation (exon or promoter), mean methylation difference (mean_delta), average methylation in PTB (mu_ptb) and FTB (mu_ftb) groups, *and* statistical significance as –log_10_(p) values (log_10_p_val). Positive mean_delta values indicate hypermethylation in PTB, while negative values represent hypomethylation.

The spatial distribution of these promoter-level methylation changes in cord blood is shown in the Circos plot (Supplementary Figure S3c). This visualization demonstrates that the epigenetic alterations observed in PTB are not restricted to specific loci but are distributed across the genome. Distinct promoter methylation patterns were observed between preterm birth (PTB) and term groups in cord blood. Promoters such as *FAM197Y2*, *IFI35*, *DKK3*, and *TSPAN11* exhibited hypermethylation in PTB samples, whereas *TMEM238*, *MGLL*, *LINC03086*, and *PER3* showed hypomethylated compared to term controls (Supplementary Figure S3d). Hierarchical clustering based on promoter methylation levels demonstrated segregation between PTB and term samples, with PTB cases forming a distinct cluster characterized by widespread promoter hypermethylation. The heatmap highlighted that *FAM197Y2*, *IFI35*, *DKK3*, and *TSPAN11* were among the most hypermethylated loci in PTB (methylation difference value of >0.55), while *TMEM238*, *MGLL*, *LINC03086*, and *PER3* were prominent among the hypomethylated regions (methylation difference value of <0.35) (Supplementary Figure S3d). A complete list of differentially methylated promoters identified in the cord blood samples is provided in Supplementary Table S4. The volcano plot highlighted *CELF2*, *IFI35*, *ISG20*, and *TULP4* among the most hypermethylated regions in PTB cord blood, while *MIR1258* and *MGLL* were prominent among the hypomethylated regions (Figure 1d).

### Differentially methylated CpG loci

In addition to region-level methylation differences, differential methylation at single-CpG resolution was assessed. Manhattan plots of individual differentially methylated CpG (DML) sites across the genome for both maternal peripheral and neonatal cord blood are presented in Figures 2a,c. A total of 25,336 CpG sites exhibited differential methylation across all maternal and neonatal samples. From these, 4,976 DMLs in maternal peripheral blood and 20,360 DMLs in cord blood passed initial filtering criteria (FDR <0.01 and absolute ∆β >0.1) and were retained for further downstream analysis. To identify the most statistically robust methylation changes, we applied a genome-wide significance threshold of −log_10_(p) >7.5. Based on this stringent cutoff, we identified 19 DMLs in maternal peripheral blood and 15 DMLs in cord blood that are considered highly significant and are discussed in detail below (Tables 3 and 4).

**FIGURE 2:**
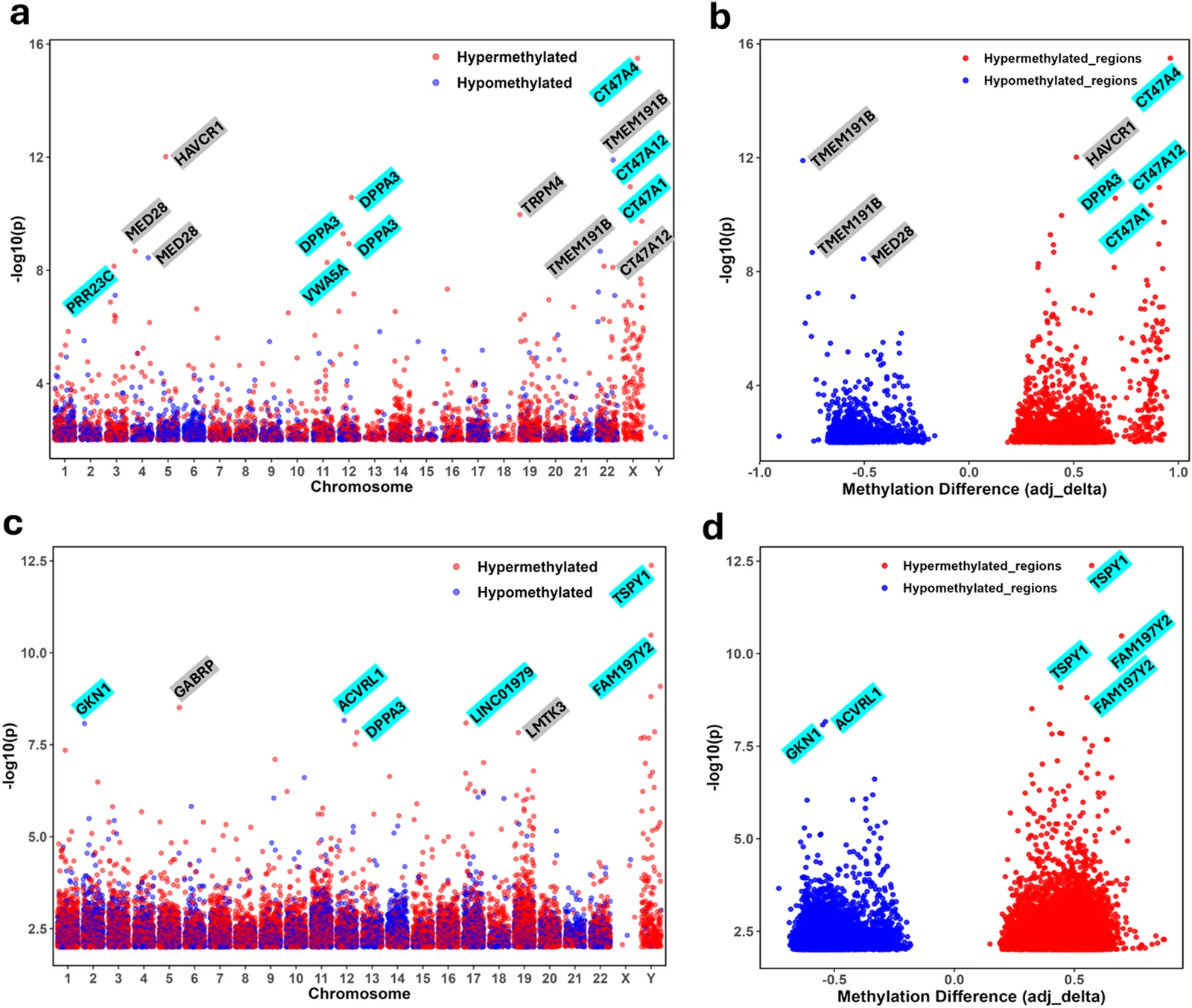
Differential methylation analysis of CpG sites in peripheral and cord blood comparing preterm birth (PTB) and full-term birth (FTB). **(a)** Manhattan plot displaying differentially methylated CpG sites in peripheral blood across chromosomal locations (x-axis). Statistical significance is represented as −log_10_(p-value) on the y-axis. Red dots indicate hypermethylation in PTB samples, while blue dots indicate hypomethylation. Genes with highly significant methylation changes [−log_10_(p) >6] include *HAVCR1*, *MED28*, *PRR23C*, *DPPA3*, *TRPM4*, *CT47A4*, *CT47A12*, *TMEM191B*, and *VWA5A*. **(b)** Volcano plot showing differentially methylated loci (DMLs) in peripheral blood. The x-axis represents the methylation difference (mean_delta), with hypermethylated loci shown in red and hypomethylated loci in blue. Statistically significant DMLs with large methylation differences (mean_delta > 0.4) include *CT47A4, HAVCR1, DPPA3, CT47A12,* and *CT47A1* [−log10(p) > 6; hypermethylated], and *TMEM191B, MED28* [−log10(p) > 6; hypomethylated]. **(c)** Manhattan plot of differentially methylated CpG sites in cord blood. Each CpG site is plotted based on its chromosomal position, with red and blue dots representing hypermethylation and hypomethylation, respectively. Genes with notable methylation differences [−log_10_(p) >7] include *TSPY1*, *FAM197Y2*, *LMTK3*, *LINC01979*, *ACVRL1*, *DPPA3*, *GABRP*, *GKN1*, *AGMAT*, and *ABCA1*, with significant methylation patterns observed in the *TSPY1* gene family on chromosome Y. **(d)** Volcano plot showing differentially methylated loci (DMLs) in cord blood, with the x-axis representing methylation differences (mean_delta). Red dots indicate hypermethylation, and blue dots indicate hypomethylation. Statistically significant DMLs with large methylation differences (mean_delta >0.3) include *TSPY1*, *FAM197Y2* [−log_10_(p) >7.5; hypermethylated] and *GKN1*, *ACVRL1* [−log_10_(p) >6.0; hypomethylated]. Genes highlighted in gray represent exonic regions, while those highlighted in turquoise indicate promoter regions.

**TABLE 3:** Top differentially methylated loci (DMLs) identified in peripheral blood samples from preterm birth (PTB) cases compared to full-term birth (FTB) controls.

Chr	Position	Mean delta	Annotation	Methylation status	log10p value	Gene Symbol
chrX	120937373	0.961	Promoter	Hypermethylated	15.496	*CT47A4*
chr5	157052557	0.512	Exon	Hypermethylated	12.018	*HAVCR1*
chr22	18529942	−0.793	Exon	Hypomethylated	11.899	*TMEM191B*
chrX	120946813	0.909	Promoter	Hypermethylated	10.955	*CT47A12*
chr12	7710434	0.698	Promoter	Hypermethylated	10.575	*DPPA3*
chrX	120942971	0.868	Promoter	Hypermethylated	10.338	*CT47A1*
chr19	49166116	0.441	Exon	Hypermethylated	9.971	*TRPM4*
chrX	120936636	0.931	Exon	Hypermethylated	9.736	*CT47A3*
chr12	7710684	0.388	Promoter	Hypermethylated	9.295	*DPPA3*
chrX	120946090	0.906	Exon	Hypermethylated	8.967	*CT47A12*
chr12	7710476	0.402	Promoter	Hypermethylated	8.942	*DPPA3*
chr4	17642193	0.404	Exon	Hypermethylated	8.681	*MED28*
chr22	18529977	-0.748	Exon	Hypomethylated	8.674	*TMEM191B*
chr4	17658459	-0.503	Exon	Hypomethylated	8.447	*MED28*
chr11	124114907	0.330	Promoter	Hypermethylated	8.279	*VWA5A*
chr3	139045129	0.328	Promoter	Hypermethylated	8.148	*PRR23C*
chr22	18518865	0.925	Promoter	Hypermethylated	8.103	*GGTLC3*
chrX	120946159	0.848	Exon	Hypermethylated	7.699	*CT47A12*
chrX	120945979	0.853	Exon	Hypermethylated	7.522	*CT47A12*

The table lists CpG sites with –log_10_(p) >7.5 and includes chromosome (Chr), genomic position (Position), mean methylation difference (mean delta), genomic annotation (e.g., promoter-TSS or exon), direction of methylation change (Hypermethylated or Hypomethylated), statistical significance (log_10_p value), and associated gene symbol. Positive mean delta values indicate hypermethylation in PTB, whereas negative values indicate hypomethylation.

**TABLE 4:** Top differentially methylated loci (DMLs) identified in cord blood samples from preterm birth (PTB) cases compared to full-term birth (FTB) controls.

Chr	Position	Mean delta	Annotation	Methylation status	log10p value	Gene symbol
chrY	9466342	0.572546	Promoter	Hypermethylated	12.38122	*TSPY1*
chrY	9465205	0.6969	Promoter	Hypermethylated	10.48107	*FAM197Y2*
chrY	9466650	0.443814	Promoter	Hypermethylated	9.089516	*TSPY1*
chrY	9465184	0.552509	Promoter	Hypermethylated	8.811588	*FAM197Y2*
chr5	170788642	0.32325	Exon	Hypermethylated	8.513615	*GABRP*
chr12	51912002	−0.53726	Promoter	Hypomethylated	8.165847	*ACVRL1*
chr17	79927533	0.39712	Promoter	Hypermethylated	8.093144	*LINC01979*
chr2	68974368	−0.548	Promoter	Hypomethylated	8.077416	*GKN1*
chrY	9467447	0.439663	Exon	Hypermethylated	7.854782	*TSPY1*
chr12	7710684	0.448189	Promoter	Hypermethylated	7.838237	*DPPA3*
chr19	48498593	0.406903	Exon	Hypermethylated	7.831976	*LMTK3*
chrY	9464777	0.543689	Promoter	Hypermethylated	7.701853	*FAM197Y2*
chrY	9466020	0.63464	Promoter	Hypermethylated	7.681619	*TSPY1*
chrY	9465149	0.637173	Promoter	Hypermethylated	7.677986	*FAM197Y2*
chr12	7710476	0.574639	Promoter	Hypermethylated	7.515505	*DPPA3*

The table lists CpG sites with –log_10_(p) >7.5. It includes chromosome (Chr), genomic position (Position), mean methylation difference (mean delta), genomic annotation (e.g., promoter-TSS or exon), direction of methylation change (Hypermethylated or Hypomethylated), statistical significance (log10p value), and associated gene symbol. Positive mean delta values indicate hypermethylation in PTB, whereas negative values indicate hypomethylation.

In peripheral blood samples of 4,976 DMLs, 16 CpG sites were hypermethylated, while 3 CpG sites were hypomethylated in PTB (Supplementary Table S5). Hypermethylated CpG sites were predominantly localized to promoter regions of genes such as *CT47A4*, *CT47A12*, *DPPA3*, *CT47A1*, *VWA5A*, *PRR23C*, and *GGTLC3*, and exonic regions of *HAVCR1*, *TRPM4*, *CT47A3*, *CT47A12*, and *MED28* (Figure 2a). These changes suggest widespread epigenetic silencing at PTB’s promoter and gene bodies. In contrast, hypomethylated CpG sites were exclusively observed in exonic regions, particularly within *TMEM191B* and *MED28*, indicating potential transcriptional activation or loss of methylation-mediated repression (Figure 2a; Supplementary Table S5). The volcano plot depicts differentially methylated loci (DMLs) in peripheral blood, contrasting PTB and FTB samples based on methylation difference (adjusted delta) and statistical significance (−log_10_ p) (Figure 2b). A clear asymmetry is evident, with significantly hypermethylated loci (red) than hypomethylated loci (blue) in PTB. Prominent hypermethylated genes, including *CT47A4*, *CT47A12*, *CT47A1*, *HAVCR1*, and *DPPA3*, show large methylation differences and high statistical significance (Table 3). In contrast, hypomethylated loci were fewer, with *TMEM191B* and *MED28* among the top candidates (Figure 2b). Consistent with these findings, the heatmap of differentially methylated CpG sites in peripheral blood demonstrates clear segregation between preterm and term groups based on methylation patterns (Supplementary Figure S4a). Hypermethylated CpG sites in PTB clustered distinctly from hypomethylated sites, reflecting widespread epigenetic shifts predominantly involving promoter and exonic regions. Hierarchical clustering revealed coordinated hypermethylation across multiple loci in PTB samples, supporting the notion of transcriptional silencing associated with preterm birth.

In cord blood samples, of 20,360 DMLs, 15 CpG sites exhibited significant differential methylation between preterm birth (PTB) and full-term birth (FTB), meeting the threshold of −log_10_ p >7.5 (Supplementary Table S6). Among these, 13 CpG sites were hypermethylated, and two sites were hypomethylated in PTB. The hypermethylated CpG sites were predominantly located in promoter regions of genes such as *TSPY1*, *FAM197Y2*, *LINC01979*, and *DPPA3*, as well as in exonic regions of *GABRP*, *LMTK3*, and *FAM197Y2* (Figure 2c). On the other hand, hypomethylated CpG sites were found in the promoter region of *ACVR1* and in *GKN1*, indicating possible transcriptional activation or decreased methylation-mediated silencing in PTB (Figure 2c; Table 4). The volcano plot visualizes differentially methylated loci (DMLs) in cord blood samples, highlighting both the magnitude (adjusted delta) and significance (−log_10_ p) of methylation differences between PTB and FTB groups (Figure 2d). A clear separation of hypermethylated (red) and hypomethylated (blue) CpG sites is observed. *TSPY1* and *FAM197Y2* exhibited the highest hypermethylation levels, while *GKN1*, *ACVRL1*, and *LAMA1* were among the top hypomethylated genes. Consistent with the locus-specific findings, the heatmap of differentially methylated CpG sites in cord blood demonstrates distinct clustering of preterm and term samples based on their methylation profiles (Supplementary Figure S4b). Hypermethylated CpG sites exhibited strong co-clustering within PTB samples, whereas hypomethylated CpG sites were predominantly associated with term samples. The hierarchical clustering analysis revealed coordinated hypermethylation across multiple genomic regions in PTB, particularly involving promoter and exonic loci, suggesting widespread epigenetic reprogramming during fetal development in preterm infants. The predominance of hypermethylated sites in PTB samples reflects enhanced epigenetic silencing mechanisms, while localized hypomethylation events may indicate selective activation of specific developmental pathways. Together, these methylation patterns in cord blood highlight fundamental alterations in the neonatal epigenome associated with PTB.

### Functional enrichment of DMR and DML associated genes

To understand the biological relevance of the methylation changes observed in both differentially methylated regions (DMRs) and loci (DMLs), we performed Gene Ontology (GO) and KEGG pathway enrichment analyses separately for maternal peripheral blood and neonatal cord blood. Genes associated with significant DMRs and DMLs in maternal blood were consistently enriched for GO Biological Processes related to neurodevelopment, cell junction organization, and ion transport, including synapse organization, axonogenesis, and mesenchyme development (Figures 3a, 4a). KEGG pathway analysis revealed strong enrichment for signaling pathways including Wnt signaling, calcium signaling, cAMP signaling, and neuroactive ligand–receptor interaction (Figures 3b, 4b). These pathways play critical roles in developmental regulation and cell communication. The recurrent enrichment of Wnt and calcium signaling across both DMRs and DMLs underscores a shared epigenetic disruption in maternal signaling pathways associated with PTB. Similarly, in cord blood, genes associated with DMRs and DMLs were enriched for GO terms related to axonogenesis, synapse assembly, regulation of membrane potential, and vascular development, reflecting a strong neurodevelopmental and circulatory signature (Figures 3c, 4c). KEGG pathways enriched in this compartment included MAPK, cAMP, Rap1, oxytocin, neuroactive ligand–receptor, and calcium signaling pathways (Figures 3d, 4d). Notably, oxytocin signaling, implicated in uterine contraction and the timing of parturition, was uniquely enriched in the cord blood DMLs, suggesting a fetal epigenetic response to labor-associated stressors. Across both compartments, calcium and neuroactive ligand–receptor signaling were consistently enriched, indicating systemic methylation changes in key developmental and neuroendocrine pathways. While shared pathways were observed, some enrichments were tissue-specific: Wnt signaling was more prominent in maternal peripheral blood, whereas oxytocin and Rap1 signaling were specific to neonatal cord blood.

**FIGURE 3:**
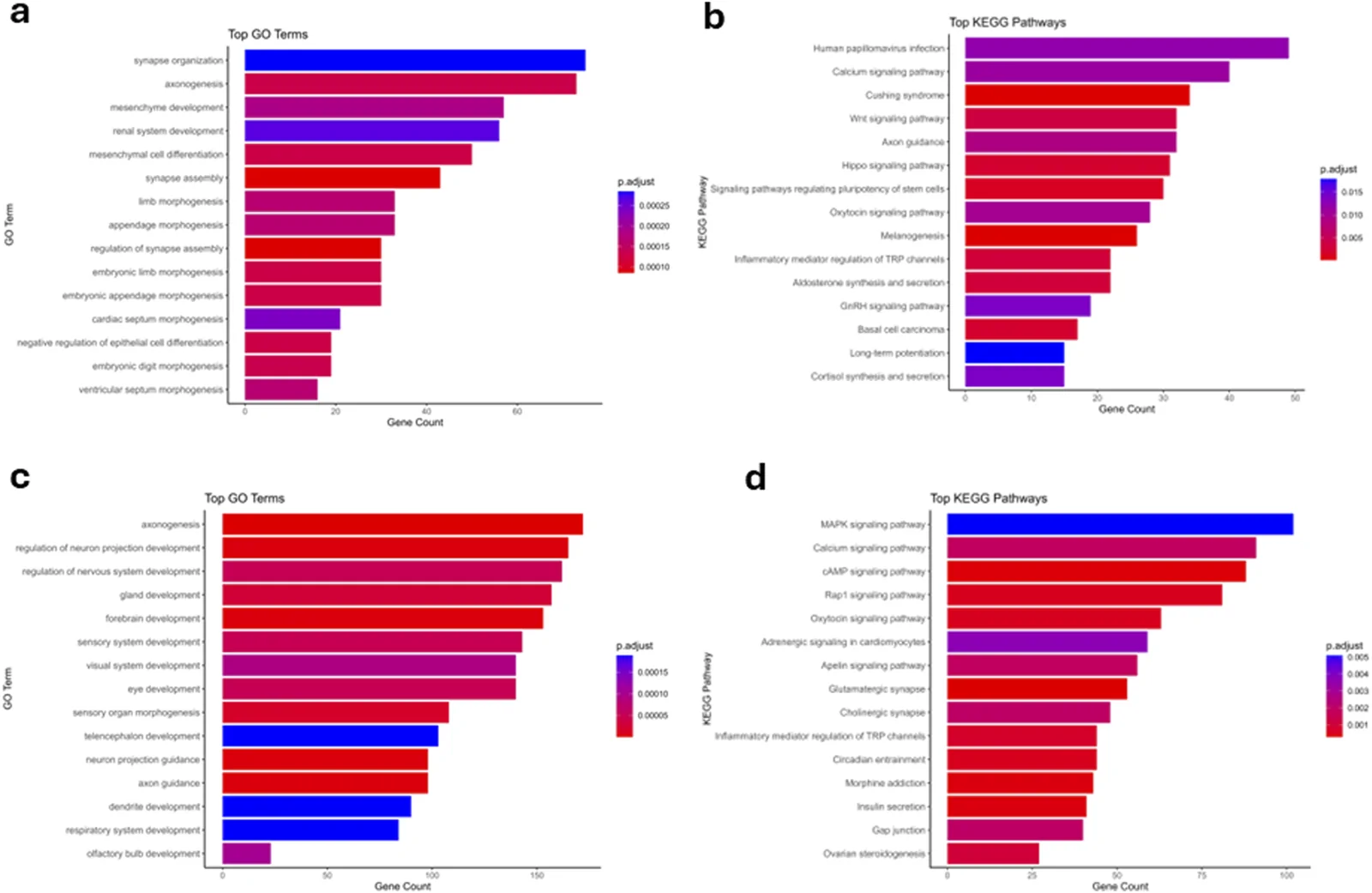
Gene ontology (GO) and KEGG pathway enrichment analyses of differentially methylated regions (DMRs) in peripheral and cord blood. **(a)** Top significantly enriched GO Biological Processes (BP) identified from genes associated with differentially methylated regions in peripheral blood. The bar plot displays the top 15 enriched GO terms ordered by gene counts. Terms such as *synapse organization*, *axonogenesis*, and *mesenchyme development* are among the most significantly enriched, highlighting key biological processes potentially impacted by methylation differences in peripheral blood. Color gradients represent the adjusted p-value (Benjamini–Hochberg corrected), with deep red indicating higher significance (lower p.adjust values) and blue representing lower significance (higher p.adjust values). **(b)** Top enriched KEGG pathways identified from genes associated with DMRs in peripheral blood. This bar plot presents the 15 most significant pathways, including *Human papillomavirus infection*, *Calcium signaling pathway*, and *Wnt signaling pathway*, which may indicate important signaling and regulatory pathways altered by differential methylation. Colors reflect statistical significance, with deeper red indicating pathways with higher significance. **(c)** Top significantly enriched GO Biological Processes (BP) identified from genes associated with differentially methylated regions in cord blood. Prominent biological processes include *axonogenesis*, *regulation of neuron projection development*, and *gland development*, suggesting a strong developmental and neural component influenced by methylation status in cord blood. The adjusted p-values are represented by the color gradient, with deep red indicating highly significant enrichment. **(d)** Top enriched KEGG pathways from genes associated with cord blood DMRs. Pathways such as *MAPK signaling pathway*, *Calcium signaling pathway*, and *cAMP signaling pathway* are highlighted, suggesting that differential methylation in cord blood prominently influences critical signal transduction pathways. Color intensity indicates the statistical significance of the enrichment, with darker shades of red corresponding to lower adjusted p-values (higher significance).

**FIGURE 4:**
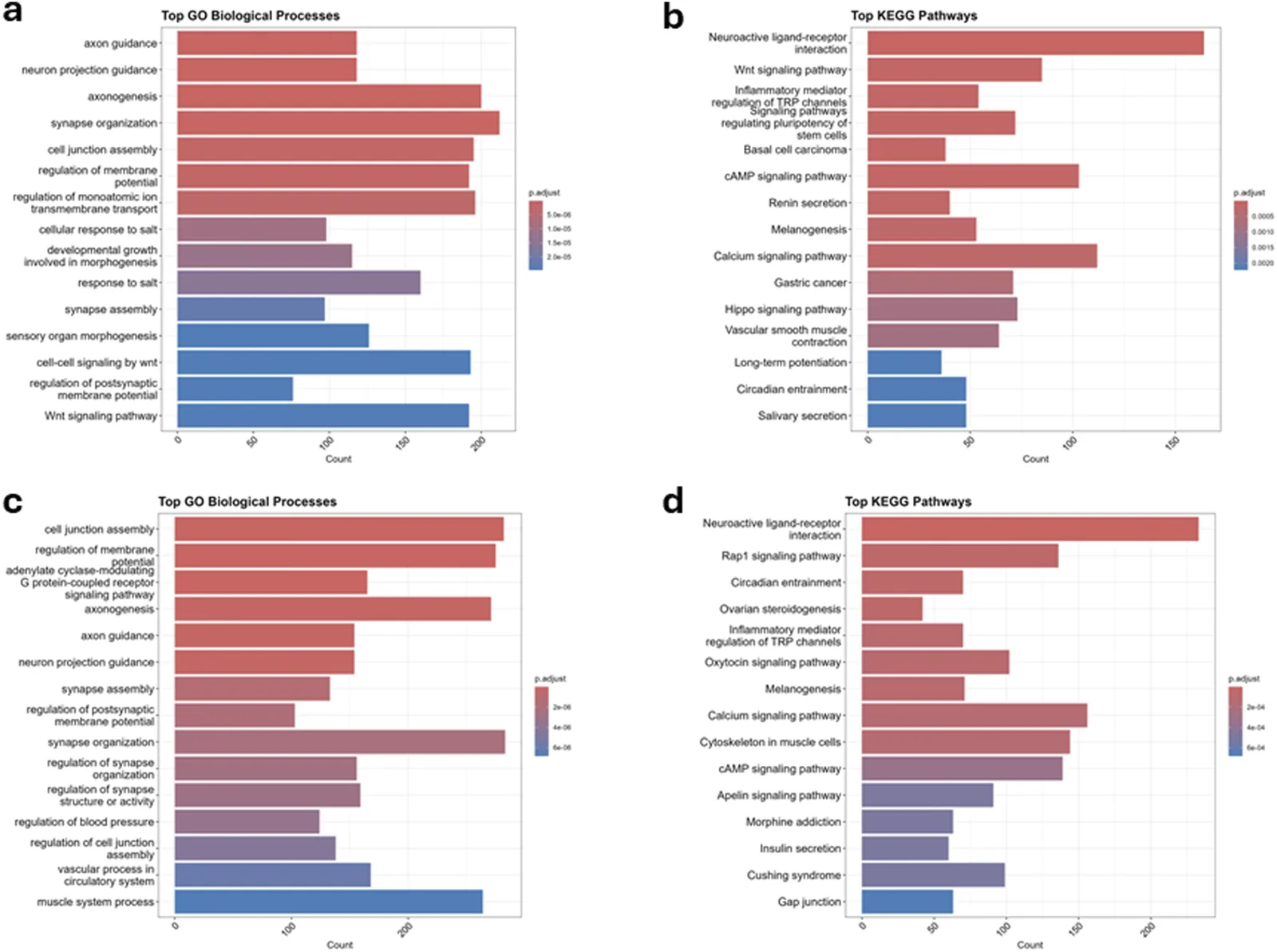
Gene ontology (GO) and KEGG pathway enrichment analysis of differentially methylated loci (DMLs) associated with preterm birth (PTB) from peripheral and cord blood. **(a)** Top significantly enriched GO Biological Processes (BP) associated with DMLs in peripheral blood. The bar plot displays the most significantly enriched GO terms related to neuronal development, synapse organization, cell junction assembly, and ion transport, among others. The x-axis represents the gene count associated with each GO term, while the color gradient represents adjusted p-values (p.adjust), with red indicating higher significance and blue indicating lower significance. **(b)** Top enriched KEGG pathways associated with DMLs in peripheral blood. Enriched pathways include neuroactive ligand-receptor interaction, Wnt signaling pathway, cAMP signaling pathway, and calcium signaling pathway, which are involved in cellular signaling and regulatory functions. The x-axis represents the gene count, and the color gradient indicates statistical significance, with deeper red signifying lower adjusted p-values (higher significance). **(c)** Top significantly enriched GO Biological Processes (BP) associated with DMLs in cord blood. Key enriched processes include cell junction assembly, regulation of membrane potential, axonogenesis, synapse assembly, and vascular processes, indicating potential roles in neurodevelopment and circulation. The x-axis represents gene count, with color intensity reflecting statistical significance. **(d)** Top enriched KEGG pathways associated with DMLs in cord blood. Enriched pathways include neuroactive ligand-receptor interaction, Rap1 signaling pathway, oxytocin signaling pathway, calcium signaling pathway, and inflammatory mediator regulation of TRP channels, highlighting key cellular signaling mechanisms. The color gradient reflects statistical significance, with red indicating higher significance and blue indicating lower significance.

### Overlap and tissue-specific patterns

To elucidate shared and tissue-specific epigenetic alterations associated with preterm birth (PTB), we performed a comparative analysis of differentially methylated loci (DMLs) between maternal peripheral blood and neonatal cord blood samples (Figure 5). This analysis revealed 120 DMLs that were common to both tissue types (overlapping DMLs), while 20,240 and 4,770 DMLs were found to be unique to cord blood and peripheral blood, respectively (Figure 5a). Among the overlapping DMLs, 91 loci demonstrated consistent hypermethylation and 28 loci exhibited consistent hypomethylation across both tissues. Only one locus showed discordant methylation patterns, indicating opposite directionality between the two compartments (Supplementary Table S7).

**FIGURE 5:**
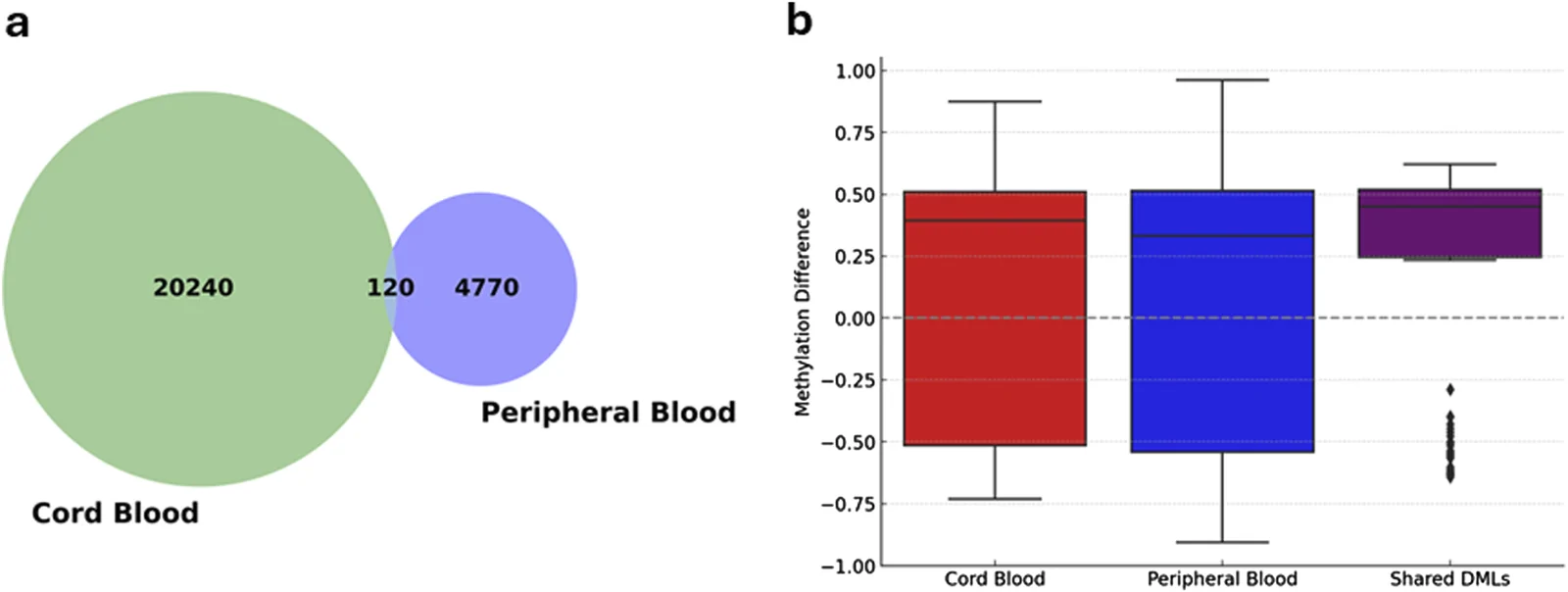
Comparison and overlap of differentially methylated loci (DMLs) in cord and peripheral blood samples. **(a)** Venn diagram displaying the overlap between significant differentially methylated loci (DMLs) identified in cord blood and peripheral blood samples. Numbers indicate the count of unique and shared DMLs, highlighting both tissue-specific and common epigenetic modifications associated with preterm birth. **(b)** Box plots illustrating methylation differences at DMLs between preterm and term groups within cord blood, peripheral blood, and shared loci between both tissues. The y-axis represents methylation differences, where positive values indicate hypermethylation and negative values indicate hypomethylation in preterm relative to term samples. Boxes represent the interquartile range (IQR), with medians indicated by horizontal lines; whiskers extend to 1.5 times the IQR, and individual dots indicate outliers. The plot underscores distinct and shared epigenetic patterns associated with preterm birth across tissue types.

The distribution of methylation differences was evaluated based on delta beta values, revealing a high degree of concordance in the directionality of methylation changes within shared DMLs between maternal and fetal samples (Figure 5b). This consistency indicates that certain PTB methylation changes are reflected in maternal and neonatal tissues. Among the most significantly shared hypermethylated genes, *DPPA3* and *ABCA1* demonstrated robust methylation differences, with delta beta values of approximately 0.40 in PB and 0.57 in CB for *DPPA3*, and 0.49 in PB and 0.43 in CB for *ABCA1* (Supplementary Table S7). In contrast, *GKN1* emerged as a prominently hypomethylated gene in both sample types, with methylation differences of approximately −0.39 in PB and −0.55 in CB. These findings demonstrate reproducible epigenetic alterations across both maternal and neonatal compartments in PTB cases.

To further characterize shared epigenetic signatures in preterm birth (PTB), we identified 14 overlapping differentially methylated regions (DMRs) that were consistently altered in maternal peripheral and neonatal cord blood samples. To assess their genomic distribution, these DMRs were annotated with associated gene symbols and chromosomal locations. Hierarchical clustering of the overlapping DMRs, visualized in a heatmap, showed that many regions clustered by chromosomal coordinates and gene associations, indicating coordinated methylation patterns across both tissue types (Figure 6a). The color scale represents the average genomic start position of each DMR, with clear groupings observed across multiple chromosomes. The clustered genes included *PLD5*, *FBXO40*, *GMNC*, *ATG9B*, *HHIP*, *PLD4*, *PIGK*, *CLEC18B*, *PCNX3*, *LHX1*, *C11orf16*, and *NINJ2*, among others. These genes were associated with overlapping DMRs across chromosomes 1, 3, 4, 7, 11, 12, 14, 16, and 17, reflecting a non-random spatial pattern of methylation alterations.

**FIGURE 6:**
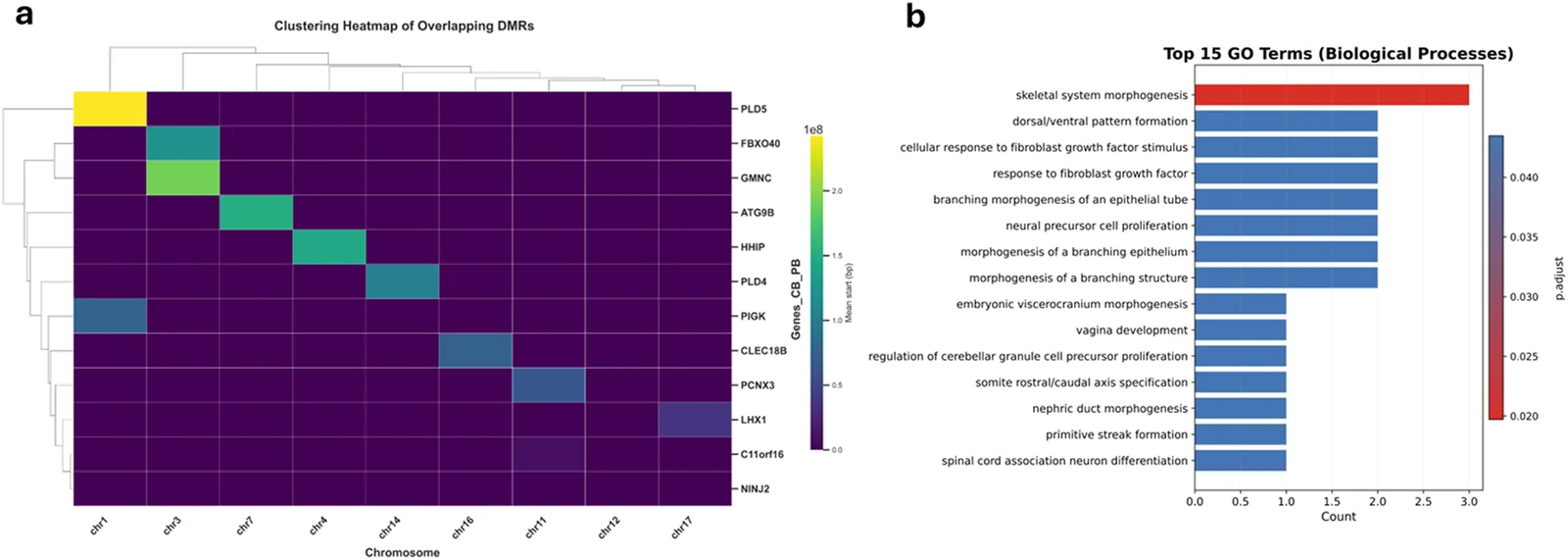
Hierarchical clustering and functional enrichment of overlapping DMRs in cord and peripheral blood. **(a)** Clustering Heatmap of Overlapping DMRs. The heatmap illustrates the hierarchical clustering of overlapping differentially methylated regions (DMRs) identified between cord and peripheral blood samples. The x-axis indicates the chromosomes containing DMRs, while the y-axis lists the associated genes. Color intensity represents the average genomic start position of each DMR, with yellow corresponding to higher genomic positions (further along chromosomes) and purple to lower genomic positions (near chromosome start sites). The dendrogram groups genes exhibiting similar methylation patterns across chromosomes, suggesting potential co-regulation or related biological functions. **(b)** Top 15 Enriched GO Biological Processes. The bar plot highlights the top 15 significantly enriched Gene Ontology (GO) Biological Process (BP) terms identified from overlapping DMRs between cord and peripheral blood. The x-axis shows the count of genes associated with each GO term, and the y-axis lists these biological processes. The color gradient (p.adjust) indicates statistical significance, with darker red reflecting more significant enrichment (lower adjusted p-values). Prominent enriched biological processes include skeletal system morphogenesis, dorsal/ventral pattern formation, and cellular response to fibroblast growth factor stimulus, emphasizing these methylation differences' potential developmental and differentiation-related functions.

To examine the biological relevance of these shared DMRs, we performed Gene Ontology (GO) enrichment analysis for associated genes (Figure 6b). The top 15 enriched GO Biological Process terms were predominantly related to developmental and morphogenetic processes, including skeletal system morphogenesis, dorsal/ventral pattern formation, and cellular response to fibroblast growth factor stimulus. These pathways are essential for embryonic development and tissue organization. Notably, such enrichment was specific to the overlapping DMRs and not observed in tissue-specific analyses, suggesting that the shared epigenetic changes may reflect a core developmental program disrupted in both maternal and fetal compartments in PTB.

In summary, methylation profiling of maternal and neonatal blood revealed widespread epigenetic alterations associated with preterm birth (PTB), with more extensive changes in cord blood. Differentially methylated genes were enriched in pathways related to neurodevelopment, immune regulation, and signaling cascades such as Wnt, calcium, and oxytocin. Shared hypermethylation of genes like *DPPA3* and *ABCA1* points to conserved developmental disruptions, while distinct tissue-specific patterns reflect the differing physiological environments of the mother and infant. Additionally, shared differentially methylated regions across maternal and neonatal blood showed coordinated methylation patterns in genes such as *PLD5*, *GMNC*, *HHIP*, and *LHX1*, spanning multiple chromosomes. These regions were uniquely enriched in developmental pathways, suggesting disruption of a core epigenetic program common to both maternal and fetal compartments in preterm birth.

## Discussion

This study presents the first comprehensive long-read methylation profiling analysis of preterm birth (PTB), evaluating matched maternal peripheral blood and neonatal cord blood samples. The results reveal widespread and tissue-specific DNA methylation changes associated with PTB. A total of 1,151 differentially methylated regions (DMRs) - 290 in maternal and 861 in cord blood and 25,336 differentially methylated loci (DMLs) were detected, highlighting extensive epigenetic remodeling. Both tissues exhibited a dominant pattern of hypermethylation, especially pronounced in cord blood. Unsupervised clustering of methylation patterns segregated PTB and term cases, indicating that maternal and fetal epigenomes carry distinct signatures of preterm birth.

In maternal blood, methylation alterations were observed across genes involved in developmental signaling, extracellular matrix (ECM) remodeling, and immune regulation. For example, hypermethylated genes such as *WNT7B*, *PSMB11*, and *MED28*, and hypomethylated genes like *MMP9*, *EXTL3*, and *SIRPB1*, highlight key biological processes previously implicated in preterm labor. These include Wnt/β-catenin signaling, immune activation, and ECM degradation, all of which are known contributors to uterine contractility, cervical ripening, and premature membrane rupture (Lindstrom and Bennett, 2005; Parets et al., 2013; Lee et al., 2016). While not all of these genes have established associations with PTB in the literature, their functional relevance in pregnancy-related pathways (e.g., T-cell development, heparan sulfate biosynthesis, NF-κB activation) supports their potential indirect roles in the pathophysiology of preterm birth (Harris et al., 2008; Ohigashi et al., 2017; Oud et al., 2017). At the promoter level, maternal PTB cases showed differential methylation in genes such as *VWA5A*, *EIF4E3*, and *ZNF571* (hypermethylated), and immune-related loci like *TNFRSF19* and *SIRPB1* (hypomethylated). These promoter methylation patterns may influence pro-inflammatory signaling cascades and are consistent with established models of inflammation-mediated labor, though functional consequences require further validation (Christiaens et al., 2008; Geng et al., 2024). RNA sequencing was not performed on maternal peripheral or cord blood samples in this study; hence, direct correlation between promoter-associated DMLs and gene expression was not assessed. Functional interpretations were instead inferred from prior literature and publicly available expression databases to suggest potential regulatory roles of the identified methylation changes. In neonatal cord blood, distinct methylation signatures were enriched in genes related to innate immunity, neurodevelopment, and vascular function. Prominent examples include *ISG20*, *IFI35*, *DKK3*, and *COL4A2* (hypermethylated), and *ALOX12*, *GKN1*, and *ACVRL1* (hypomethylated). These genes are involved in interferon-stimulated responses, Wnt pathway modulation, vascular stability, and trophoblast function–key biological processes essential for fetal adaptation and placental development (de Vries et al., 2001; Junus et al., 2012; Fahlbusch et al., 2013; Cappelletti et al., 2017; Hausman-Kedem et al., 2021; Akram et al., 2022). At the locus level, maternal blood showed widespread hypermethylation across autosomes and the X chromosome, including genes such as *DPPA3*, *TRPM4*, and *CT47A4*, while *TMEM91B* was hypomethylated. Cord blood displayed strong hypermethylation on the Y chromosome—particularly *TSPY1* and *FAM197Y2* pointing to potential fetal sex-specific epigenetic signatures. Shared methylation changes in *DPPA3* and *ABCA1* across maternal and fetal compartments suggest systemic epigenetic alterations that may reflect intrauterine stress responses or early-life programming effects (Hajkova, 2011; Houde et al., 2013).

Given the higher incidence of PTB among male infants, infant sex was included as a covariate in DMR and DML analyses to control for sex-related epigenetic variation. While this enabled analysis of bulk methylation differences between PTB and FTB, the limited sample size precluded sex-stratified analyses. Future studies with larger cohorts are needed to uncover sex-specific epigenetic signatures contributing to PTB susceptibility.

Our findings expand on previous epigenome-wide association studies (EWAS). A notable study by Hong et al. identified 45 CpG loci in maternal blood associated with PTB using an Illumina 450K array in a prospective birth cohort of African-American women but found no overlap in cord blood methylation at those sites (Hong et al., 2018). As epigenetic marks, particularly DNA methylation, are known to vary across populations and with ancestry, the findings from Hong et al. may reflect ancestry-specific signatures of PTB (Hong et al., 2018). Accordingly, our study is the first long-read methylation profiling of PTB in a Middle Eastern cohort, addressing a key gap in population-representative epigenetic research. By leveraging long-read sequencing, we achieved deeper CpG coverage and identified additional loci, including *DPPA3* and *ABCA1*, that were not captured using 450K arrays (Hong et al., 2018). These data highlight the added value of long-read methylation profiling in uncovering novel epigenetic contributors to PTB. Studies of placental methylation have shown mixed results. For instance, Parets et al. reported that *MMP9* hypomethylation in placental tissues correlates with gestational age, mirroring our findings in maternal blood (Parets et al., 2013). On the other hand, Brockway et al. observed minimal methylation differences in idiopathic PTB placentas and proposed the concept of “placental hypermaturity,” wherein PTB placental methylation resembles term profiles (Brockway et al., 2023). This discrepancy may reflect the limitation of placental tissue in capturing systemic or immune-related perturbations, which are more readily detected in blood. Transcriptomic studies further support our methylation findings. Akram et al. conducted transcriptomic analysis on preterm versus term placental samples using RNA-seq and identified dysregulation in Wnt and oxytocin signaling pathways in PTB placentas, including differential expression of *WNT7A*, *BAMBI*, and *ITPR2*, which we also observed as enriched pathways in our methylome data (Akram et al., 2022). These shared features between transcriptomic and epigenomic studies strengthen the biological relevance of Wnt and calcium signaling pathways in PTB pathophysiology.

Functional enrichment analyses revealed consistent disruption in several key biological pathways. Genes involved in Wnt signaling (*WNT7B*, *DKK3*), Hedgehog signaling (*HHIP*), inflammation and interferon signaling (*ISG20*, *IFI35*, *SIRPB1*, *TNFRSF19*), and extracellular matrix (ECM) remodeling (*MMP9*, *COL4A2*) were significantly altered. Enriching the neuroactive ligand-receptor interaction pathway suggests an epigenetic imprint of neuroendocrine signaling, an axis known to mediate maternal stress responses and influence gestational timing (Mbiydzenyuy et al., 2022). Such epigenetic dysregulation may accelerate parturition by tipping the balance toward uterine activation, inflammation, and membrane weakening (Menon et al., 2012; Zakar and Paul, 2020; Vidal et al., 2022). These findings carry substantial translational and clinical implications. Identifying robust epigenetic markers in maternal blood raises the possibility of developing predictive biomarker panels for PTB risk stratification. However, to ensure their clinical utility, these methylation biomarkers particularly *ABCA1* and *DPPA3*, must be validated in larger, ethnically diverse, and longitudinal cohorts. Such validation is essential to confirm their robustness and generalizability across populations. A targeted methylation panel including *WNT7B*, *MMP9*, *SIRPB1*, and *ABCA1*, among others, could enhance the predictive accuracy of existing risk models and enable earlier clinical intervention (Hong et al., 2018). Moreover, detecting shared methylation markers like *ABCA1* in maternal and fetal compartments opens the door to non-invasive testing via cell-free DNA in maternal plasma. Cell-free DNA methylation profiling has already shown promise in other pregnancy complications, and its application in PTB could provide a minimally invasive tool for early detection (Baetens et al., 2024; van Vliet et al., 2025). Cord blood methylation signatures also hold prognostic value. Genes such as *ALOX12* and *ABCA1*, which are involved in inflammatory and metabolic regulation, may affect neonatal lung development and immune maturation (Kotlyarov, 2021; Pernet et al., 2023). These methylation marks may serve as biomarkers for identifying neonates at elevated risk for adverse outcomes, though their regulatory relevance remains to be validated by future expression studies. Regarding therapeutic development, our findings support targeting specific epigenetically altered pathways. For example, the hypermethylation of *DKK3* and *WNT7B* reinforces the potential utility of Wnt pathway inhibitors in managing PTB risk. Likewise, our observation of oxytocin-calcium signaling pathway enrichment supports the rationale for using oxytocin receptor antagonists (e.g., atosiban) in tocolytic therapy (Kim et al., 2019). Inflammation-modulating drugs (e.g., anti-TNF agents) might also be considered in cases where immune-related methylation signatures dominate. Finally, the findings underscore the importance of addressing modifiable maternal exposures, such as smoking, infection, and stress, that influence epigenetic programming. Public health interventions to improve the intrauterine environment could help reduce the incidence of PTB by mitigating adverse epigenetic shifts (Knight and Smith, 2016; Jain et al., 2022).

A key limitation of this study is the inclusion of PTB cases with heterogeneous clinical backgrounds, including gestational diabetes mellitus (GDM), infection, and maternal obesity. These cases were retained to reflect real-world PTB variability, but such conditions may have influenced the observed methylation profiles and confounded interpretation. Correction for cell-type heterogeneity in peripheral maternal whole blood was not performed in this study. Genomic DNA was extracted from whole blood, which contains a heterogeneous population of nucleated immune cells, each with distinct DNA methylation profiles. This study did not include targeted analysis of imprinted genes or repetitive elements, as comparisons focused on PTB vs. FTB within each tissue type without prioritizing specific genomic features. Future studies should investigate these loci and utilize deconvolution methods compatible with long-read platforms to further enhance our understanding of epigenetic regulation in PTB.

In conclusion, this study provides a comprehensive epigenetic landscape of preterm birth (PTB) through high-resolution long-read DNA methylation profiling of paired maternal peripheral and neonatal cord blood. We identified extensive and tissue-specific epigenetic remodeling, with a predominant hypermethylation pattern, especially in cord blood from PTB cases. Differential methylation was observed in genes associated with extracellular matrix remodeling (*MMP9*, *COL4A2*), developmental signaling (*WNT7B*, *DKK3*, *LHX1*), immune modulation (*ISG20*, *IFI35*, *TNFRSF19*), and lipid metabolism (*ABCA1*), highlighting key physiological processes disrupted in early parturition. Importantly, a subset of concordant methylation changes, including *DPPA3* and *ABCA1*, was shared between maternal and fetal compartments, suggesting coordinated or systemic epigenetic responses that may reflect intrauterine stress or transgenerational regulation. These shared signatures and broader compartment-specific alterations provide new insights into the maternal-fetal epigenetic interface in PTB. Long-read sequencing enabled precise mapping of differentially methylated loci and regions (DMLs and DMRs), offering a level of resolution not achievable with prior array-based methods. The findings reveal pathway-level disruptions across immune, hormonal, neuroendocrine, and developmental axes, supporting the multifactorial nature of PTB pathogenesis. These results establish a valuable foundation for future biomarker discovery and mechanistic studies. Identifying robust and clinically relevant methylation signatures opens promising avenues for developing predictive assays, informing early interventions, and guiding personalized obstetric care to improve maternal and neonatal outcomes in PTB.

## Future directions

This study establishes a framework for advancing the clinical and mechanistic utility of DNA methylation biomarkers in PTB. Future research should validate the identified DMLs and DMRs particularly shared maternal-fetal markers such as *ABCA1* and *DPPA3* in larger, ethnically diverse, and longitudinal cohorts to ensure generalizability and robustness of these markers. Expanding the sample size is essential not only to ensure statistical power but also to account for biological and clinical heterogeneity across PTB cases. Robust validation across larger cohorts is a critical step toward developing reliable and generalizable methylation-based biomarkers of PTB risk. Determining their temporal dynamics and predictive power across gestation will be critical for clinical translation. Integrating methylation data with transcriptomic, proteomic, and metabolomic profiles will enable systems-level insights into how epigenetic alterations influence gene expression, immune activation, extracellular matrix remodeling, and neuroendocrine signaling during pregnancy. Functional studies using *in vitro* models (e.g., trophoblasts, decidual cells) and *in vivo* PTB models are needed to clarify the causal roles of key epigenetically regulated genes, such as *ISG20*, *WNT7B*, and *TNFRSF19*. Furthermore, investigation into the influence of modifiable maternal exposures, such as infection, stress, and nutritional status, on DNA methylation will inform prevention strategies. The potential for developing non-invasive methylation-based assays using cell-free DNA should also be explored, particularly given shared maternal-fetal signatures. Given the long-read sequencing platform used in this study, future work should also leverage its capacity to detect allele-specific methylation (ASM). This could uncover parent-of-origin effects, imprinting disruptions, and allele-biased epigenetic regulation that may be implicated in PTB risk. Finally, assessing the reversibility of PTB-associated methylation changes could open avenues for epigenetic interventions, including nutritional supplementation or pathway-targeted therapies. Together, these directions will advance the development of predictive, preventive, and personalized approaches to reduce the burden of preterm birth (Al-Dewik et al., 2022; Al-Dewik and Qoronfleh, 2019; Zhai et al., 2023).

## Data Availability

The datasets presented in this study are available in the GitHub repository: https://github.com/NaderHMC-lab/dna-methylation-analysis.
