# Supplementary material for: Epigenetic profiling of preterm birth: a dual-tissue methylation patterns using long-read sequencing
Source: Front Epigenet Epigenom. 2025 Dec 5;3:1644521. doi: 10.3389/freae.2025.1644521 (PMC13639308; doi:10.3389/freae.2025.1644521)
Supplement: Supplementary file 1 [file DataSheet1.docx]

**Epigenetic Profiling of Preterm Birth: A Dual-Tissue Methylation Patterns Using Long-Read Sequencing Running head: Differential Methylation Analysis in Preterm Birth**

BalaSubramani Gattu Linga^1,2^*, Faisal E. Ibrahim^1-3*^, Aleem Razzaq^1,2*^, Muthanna Samara^4^, Jameela Roshanuddin^1,5^ Hind H. Adi1^1,6^, Aseel Al-Dewik^1-7^, Ayla J. Ahmedoglu^1,6^, Rand Hamdan^1,6^, Amal E.I. Ahmed^1,8^, M. Walid Qoronfleh^9^, Hatem Zayed^10^, Dua Elshiekh^10^, Mona Ellaithi^11^, Mohamed Alsharshani^12^, Palli Valapila Abdulrouf^1,13^, Thomas Farrell^1,14,15^, Bader Kurdi^16^, Ghassan abdo^16^, Hilal Al Rifai^16^, Nader Al-Dewik^1-3,5,16-18Ψ^

**Figure S1. Chromosomal Distribution of Differentially Methylated Regions (DMRs) in Peripheral and Cord Blood Samples.** Panels illustrate the genomic locations of significant DMRs (red triangles) identified in maternal peripheral blood (a) and neonatal cord blood samples (b). The x-axis denotes chromosome length in megabases (Mb), and the y-axis lists individual chromosomes. Horizontal black bars represent chromosome lengths, facilitating comparisons of DMR distribution across chromosomes. Variations in DMR density and positioning highlight tissue-specific methylation patterns between peripheral and cord blood samples.

**Figure S2. Differentially Methylated Genes (DMGs) and promoters (DMPs) identified in Preterm Birth (PTB) condition in Maternal Peripheral Blood Tissue.** (a) Circos plot depicting significant DMGs identified in maternal peripheral blood. The outermost ring represents human chromosomes (HG38 reference genome) with labeled and color-coded chromosome ideograms. The second ring annotates significantly differentially methylated genes at their genomic positions. The third ring provides a heatmap illustrating methylation differences between PTB and FTB infants. The innermost scatter plot indicates hypermethylated (red) and hypomethylated (blue) genes. Internal red and blue lines suggest potential genomic interactions or co-methylation relationships between genes. (b) Heatmap comparing methylation patterns of the top 10 hypermethylated and hypomethylated genes in preterm (left) maternal peripheral blood samples *versus* term infants (right). The color gradient (red: hypermethylation, blue: hypomethylation) highlights significant methylation variations. Notably hypermethylated genes include *AGRN, MRGPRB, NPWBR1, MLYCD*, *HOXD13*, and *IL15R*, while *PSMB11, SMPD1, TBC1D10A, CLU6*, and COX6A2 exhibit hypomethylation. (c) Circos plot depicting significant DMPs in peripheral blood. The outermost ring represents the human chromosome ideogram (HG38 reference genome), with chromosomes labeled and color-coded. The second ring annotates gene names at respective genomic locations. The third ring provides a heatmap indicating the magnitude of differential methylation between PTB and FTB infants. The innermost scatter plot differentiates hypermethylated (red) and hypomethylated (blue) promoters. Internal red and blue connection lines suggest potential regulatory interactions or co-methylation patterns between promoter regions. (d) Heatmap of top 10 hypermethylated and hypomethylated promoters in maternal peripheral blood, comparing PTB and FTB infants. The color scale (blue: hypomethylation; red: hypermethylation) highlights methylation differences. Notably hypermethylated promoters in PTB include *MIR3667HG, MIR10522, PTPRH, OSMR,* and *SLC6A12*; significantly hypomethylated promoters include *RNF213, TUBGCP6, TNFRSF19, SDK1*, and *ZNF571*, suggesting their involvement in PTB-related epigenetic regulation.

**Figure S3. Differentially Methylated Genes (DMGs) and promoters (DMPs) identified in Preterm Birth (PTB) condition in Neonatal Cord Blood Tissue.** (a) Circos plot representing significantly differentially methylated genes in neonatal cord blood samples. The outermost ring represents the human chromosome ideogram (HG38 reference genome), with chromosomes labeled and color-coded. The second ring annotates gene names at respective genomic locations. The third ring provides a heatmap indicating the magnitude of differential methylation between PTB and FTB infants. The innermost scatter plot differentiates hypermethylated (red) and hypomethylated (blue) promoters. Internal red and blue connection lines suggest potential regulatory interactions or co-methylation patterns between promoter regions. (b) Heatmap of the top 10 hypermethylated and hypomethylated genes in neonatal cord blood comparing PTB and term-born infants. Genes exhibiting significant hypermethylation in PTB include *COL4A2, ISG20, ALOX12*, and *RBM15B*, while *TCAF2, TPM1*, and *RBM15B* are significantly hypomethylated. These findings suggest crucial roles for these genes in PTB-associated epigenetic regulation. (c) Circos plot of significant DMPs in neonatal cord blood. The outermost ring represents the human chromosome ideogram (HG38 reference genome), with chromosomes labeled and color-coded. The second ring annotates gene names at respective genomic locations. The third ring provides a heatmap indicating the magnitude of differential methylation between PTB and TB infants. The innermost scatter plot differentiates hypermethylated (red) and hypomethylated (blue) promoters. Internal red and blue connection lines suggest potential regulatory interactions or co-methylation patterns between promoter regions. (d) The heat map displays the top 10 hypermethylated and hypomethylated promoters in cord blood, grouped by methylation similarity between PTB and FTB infants. Genes demonstrating hypermethylation in PTB include *TMEM238, IFI35, DKK3, TSPAN11*, and *GIMAP5*, whereas *MGLL, LINC03086, PER3, MIR1258*, and *NT5C3B* exhibit hypomethylation, implicating their possible roles in epigenetic pathways related to preterm birth.

**Figure S4. Heatmaps of Differentially Methylated CpG Sites (DMLs) in Maternal Peripheral Blood and Neonatal Cord Blood Samples.** (a) Heatmap of differentially methylated CpG sites in maternal peripheral blood samples from preterm and full-term groups. The top differentially methylated sites are displayed, including both hypermethylated and hypomethylated CpG sites. Each row represents a CpG site associated with a specific gene, with chromosomal locations in parentheses. Methylation levels are represented by a color gradient, where blue indicates hypomethylation (low methylation) and red indicates hypermethylation (high methylation). Hierarchical clustering was applied to CpG sites to reveal distinct methylation patterns between preterm and full-term groups. (b) Heatmap of differentially methylated CpG sites in neonatal cord blood samples from preterm and term groups. The visualization follows the same format as in (a), with CpG sites linked to specific genes, chromosomal locations in parentheses, and methylation levels depicted by the same color gradient. Hierarchical clustering highlights epigenetic differences between preterm and term samples. The color scale on the right of each heatmap quantifies methylation levels, ranging from 0 (hypomethylation) to 1 (hypermethylation).
